# Supplementary figures and images for: Sarm1 knockout protects against early but not late axonal degeneration in experimental allergic encephalomyelitis
Source: PLoS One. 2020 Jun 25;15(6):e0235110. doi: 10.1371/journal.pone.0235110 (PMC7316289; doi:10.1371/journal.pone.0235110)

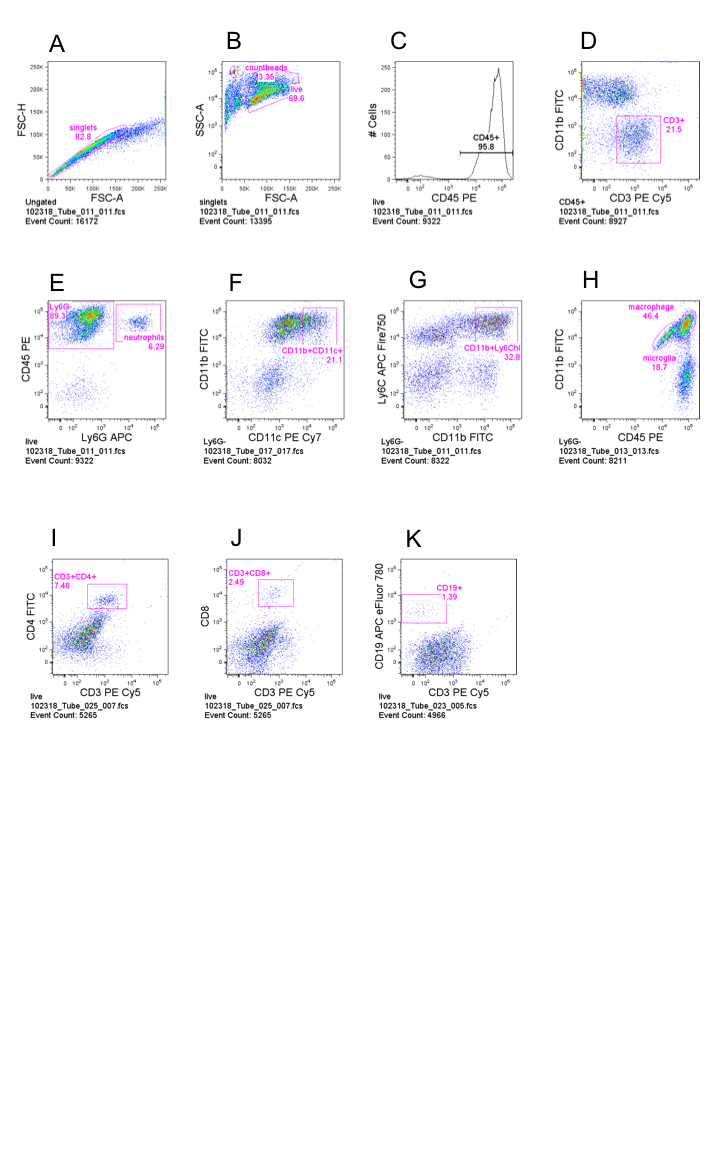

Supplement: S1 Fig — Representative plots of FACS analysis to enumerate CNS inflammatory cells from thoracolumbar cords of EAE mice are shown. A) Gating for singlets B) Gating for Count beads and live cells. C) Total CD45+ (leukocyte) gating. D) CD45+CD3+ (T cell) gating. E) CD45+Ly6G+ (neutrophil) gating. F) Ly6G-CD11b+CD11c+ (CD11b+ dendritic cell) gating. G) Ly6G-CD11b+Ly6Chi (Ly6C high monocyte/macrophage) gating. H) Ly6G-CD11b+CD45int (microglia) and Ly6G-CD11b+CD45hi (macrophage) gating. I) CD3+CD4+ (CD4 T cell) gating. J) CD3+CD8+ (CD8 T cell) gating. K) CD19+ (B-cell) gating. (TIF) [file pone.0235110.s001.tif]
