# Supplementary material for: Sarm1 knockout protects against early but not late axonal degeneration in experimental allergic encephalomyelitis
Source: PLoS One. 2020 Jun 25;15(6):e0235110. doi: 10.1371/journal.pone.0235110 (PMC7316289; doi:10.1371/journal.pone.0235110)
Supplement: Attachment — Submitted filename: Response to PLOS ONE Reviewers.docx [file pone.0235110.s002.docx]

Response to PLOS ONE reviewer comments

PONE-D-20-10362

Dear PLOS ONE Reviewers

Thank you for your constructive comments. The manuscript has been revised in response to your comments.

Reviewer #1

1. *The phenotypic data currently available in the supporting information is difficult to follow. A more extensive figure legend would be useful to be certain that the reader understands the data presented, including what populations are shown in each section and what gates are applicable to individual graphs*

A more extensive figure legend has been provided for Supporting information S1. Fig 1.

2. *The authors conclude that " SARM1 activation is not the critical trigger for irreversible axonal injury in CNS inflammatory disorders" and suggest that SARM1 involvement in axonal loss is insult dependent. However the authors do not note that this intermediate outcome, protection from early loss with slightly later loss of axon integrity is not typical of previous reports regarding SARM1 activity. The finding that SARM1 appears to block axonal damage at the peak of disease could, and should be discussed in greater detail. This will probably also have to modify the authors statement that "There was a significant inverse correlation between SMI-31+ axonal density and cumulative clinical scores, supporting the clinical relevance of axonal loss to neurological dysfunction in EAE."*

The statement “SARM1 activation is not the critical trigger for irreversible axonal injury in CNS inflammatory disorders” has been removed in the revised manuscript. An alternative explanation for the finding that *Sarm1* deletion protects early but not late axonal degeneration has been proposed in the discussion, based on mechanistic distinction between acute and chronic axonal degeneration in EAE and multiple sclerosis. The manuscript was also revised to qualify the statement regarding the inverse correlation between SMI-31+ axonal density and cumulative clinical scores to specify that this correlation applies to a late time point in the course of EAE.

3. *The authors note that the lack of examination of demyelination is a limitation. It is worth*

*discussing how the results reported may impact myelination/demyelination.*

The manuscript has been revised to discuss possible impact on myelination/demyelination: “SARM1 function is not known to be involved in myelin formation or oligodendrocyte survival, and thus *Sarm1* deletion is unlikely to directly affect myelination. However, we have not excluded the possibility that early axonal protection may indirectly affect remyelination in EAE. Further work is needed to ascertain the impact of Sarm1 deletion on demyelination/ remyelination in the setting of CNS inflammation.”

Reviewer #2

1) *The authors show that axons are well protected for about 2 weeks, and then degenerate in the SARM1 Ko during EAE. This is almost the identical time course to the protection afforded by SARM1 following axotomy in vivo. Axons survive for about 2 weeks and then degenerate in a SARM1- independent manner, likely due to the absence of cell-body derived factors. In EAE, it is very likely that the immune attack causes severing of axons and so it quite similar to axotomy. Do the authors see evidence for axon severing in their wild type mice? This would also explain the inability of SARM1 KO to change the disease course. Severed axons obviously can't contribute to function. The authors should acknowledge that all their data can be explained by the occurrence of axon severing in the EAE model.*

The manuscript has been revised to acknowledge the consequence of axonal transection: “Alternatively, the results of the study may indicate that *Sarm1* deletion simply delays the perhaps inevitable axonal degeneration that follows axonal transection, similar to that seen following axotomy”

2) *Based on their EAE results, the authors making sweeping conclusions about the role of SARM1 in CNS neuroinflammatory disease. This is dramatically overstating what can be concluded from this model. While it is clear that the EAE model is a reasonable model of the inflammatory component of MS, it is not known if EAE models the neurodegenerative component of MS. Indeed, based on the time course it seems quite unlikely. Hence, it is inappropriate to conclude that SARM1 is not involved in CNS neuroinflammatory disorders. These statements should be removed, and the limitations of the EAE model for studying the neurodegenerative component of MS should be clearly acknowledged.*

The manuscript has been revised to remove the statement “SARM1 activation is not the critical trigger for irreversible axonal injury in CNS inflammatory disorders”. An alternative explanation has been proposed in the discussion based on mechanistic distinction between acute and chronic axonal degeneration in EAE and multiple sclerosis. Furthermore, the manuscript has been revised to acknowledge the limitations of EAE in modeling neurodegeneration of multiple sclerosis.

3) *The title states that SARM1 KO does not "protect against axonal degeneration" in EAE. However the data clearly show that it does "protect," even if it doesn't permanently block. Again, this is identical to the result with axotomy--nothing can permanently block degeneration of a severed axon. The title should be changed to reflect that SARM1 Ko does protect against axon degeneration in EAE, albeit not indefinitely.*

In response to reviewer comments, the manuscript title has been revised to “Sarm1 knockout protects against early but not late axonal degeneration in experimental allergic encephalomyelitis”. In addition the conclusion has also been revised to state early but not late protection of axonal degeneration in Sarm1 KO in EAE.
