# Supplementary material for: Sarm1 knockout protects against early but not late axonal degeneration in experimental allergic encephalomyelitis
Source: PLoS One. 2020 Jun 25;15(6):e0235110. doi: 10.1371/journal.pone.0235110 (PMC7316289; doi:10.1371/journal.pone.0235110)
Supplement: Attachment — Submitted filename: Response to reviewers 060220.docx [file pone.0235110.s003.docx]

PONE-D-20-10362R1

Response to Reviewers

Reviewer #2: *The authors made most of the requested changes but missed a couple of spots. In addition there are a few missing references. 1) I had asked the authors not claim that there findings were valid for all CNS inflammatory disorders, but just for this one model. They made the changes most places, but missed line 451 and line 471. Please change to "model of" here as well. 2) The authors discuss the potential role of SARM1 in the immune system, but do not mention an important new paper that suggests most of the referenced studies are an artifact of the genetic background in the line used by the authors. The authors should acknowledge this issue and reference the paper (Uccellini 2020). 3) The authors mention one study that did not fine a role for SARM1 in an ALS model, but leave out another study that did find a role (White 2019). In fact this paper was similar to the author's work, showing some axonal protection but not behavioral protection. This second study should be acknowledged.*

1) The manuscript has been revised in response to reviewer comments.

We agree with the reviewer and line 471 has been changed in the revised manuscript to:

471 “... degeneration in this model of immune-mediated CNS inflammatory disorder.”

However, we are confounded by the reviewer’s request for change in line 451 which reads as follows in the resubmitted manuscript:

451 “*Sarm1* deletion conferred early, but not long-term, axonal protection in EAE.”

There is no claim to validity for CNS inflammatory disorders in general in line 451, therefore we are at a loss as to how that should to be changed.

2) The paper by Uccellini et al reporting that background effects in part explain some of the previously reported effects of SARM1 on immune response has been acknowledged and referenced in the revised Discussion.
3) In addition, the paper by White et al was also acknowledged in the revised Discussion.
